# Post-tuberculosis Destroyed Lung Syndrome in an Indian Male: A Case Report

**DOI:** 10.7759/cureus.56847

**Published:** 2024-03-24

**Authors:** Sankalp Yadav

**Affiliations:** 1 Medicine, Shri Madan Lal Khurana Chest Clinic, New Delhi, IND

**Keywords:** post-pneumonectomy, hemoptysis, mycobacterium tuberculosis (mtb), destroyed lung syndrome (dls), destroyed lung

## Abstract

The term "destroyed lung" signifies the complete degradation of lung tissues, typically due to chronic or recurring lung infections, with tuberculosis often identified as a primary culprit. This condition, when occurs after tuberculosis and is known as post-tubercular destroyed lung syndrome, poses considerable difficulties, especially in areas where tuberculosis is prevalent. This paper outlines a case study involving a 50-year-old Indian man afflicted with destroyed lung syndrome. Despite having undergone tuberculosis treatment three years earlier, the patient exhibited symptoms such as a dry cough, coughing up blood, and difficulty breathing. A comprehensive clinical evaluation and radiological assessments confirmed the diagnosis of destroyed lung syndrome, leading to the commencement of appropriate treatment.

## Introduction

Pulmonary tuberculosis, caused by the bacillus *Mycobacterium tuberculosis*, has afflicted humanity since ancient times [1]. In India, it stands as one of the most prevalent infectious lung diseases, contributing significantly to both mortality and morbidity rates [2]. Tuberculosis manifests in various thoracic presentations, ranging from consolidations, nodules, and cavitations to mediastinal adenopathy, pleural effusion, and diffuse endobronchial disease resembling bronchial asthma [3].

The medical literature acknowledges "destroyed lung" as a complication arising from pulmonary tuberculosis, characterized by severe damage to both pleural and parenchymal lung tissues [4]. This damage includes cavitation, bronchiectasis, reduced lung volume, and displacement of the mediastinum toward the affected lung [3]. Tuberculosis is widely recognized as the primary cause of this condition, either as a result of an initial infection or reinfection [5]. Despite its significance, there is a scarcity of literature addressing this condition, and precise prevalence rates remain unknown [4].

This report presents a case study involving a 50-year-old Indian man diagnosed with destroyed lung syndrome. Despite undergoing tuberculosis treatment three years prior, the patient experienced remarkable symptoms, prompting a diagnostic examination and management.

## Case presentation

A 50-year-old Indian male from a low-income background presented with chief complaints of dry cough, hemoptysis, and dyspnea. The cough had been intermittent, without expectoration, persisting for nearly six months. Over the past week, he experienced four episodes of hemoptysis, with the most recent occurring on the day of presentation. Previously, he had sought advice from local physicians for these symptoms and was given over-the-counter antitussive syrup containing dextromethorphan. Moreover, the patient experienced progressive dyspnea at rest over a two-week period. Initially, he reported grade 1 dyspnea according to the modified Medical Research Council dyspnea scale, which worsened to grade 4 over six months. There were no reported fevers, loss of appetite, night sweats, or weight loss.

His history was remarkable for drug-sensitive tuberculosis three years ago. He had completed his treatment at that time, and the outcome was declared cured in the national data portal. Personal history was notable for the use of tobacco chewing (1 pack of 50 gm in three days for 24 years).

Upon general examination, the patient presented with a lean, ectomorphic physique. Vital signs were within normal limits, including a pulse rate of 89 beats per minute, arterial blood pressure of 130/80 mmHg, and a respiratory rate of 27 breaths per minute. Oxygen saturation (SpO_2_) was 96% at rest but dropped to 89% following physical exertion. No signs of pallor, icterus, cyanosis, lymphadenopathy, or edema were noted.

**Figure 1:**
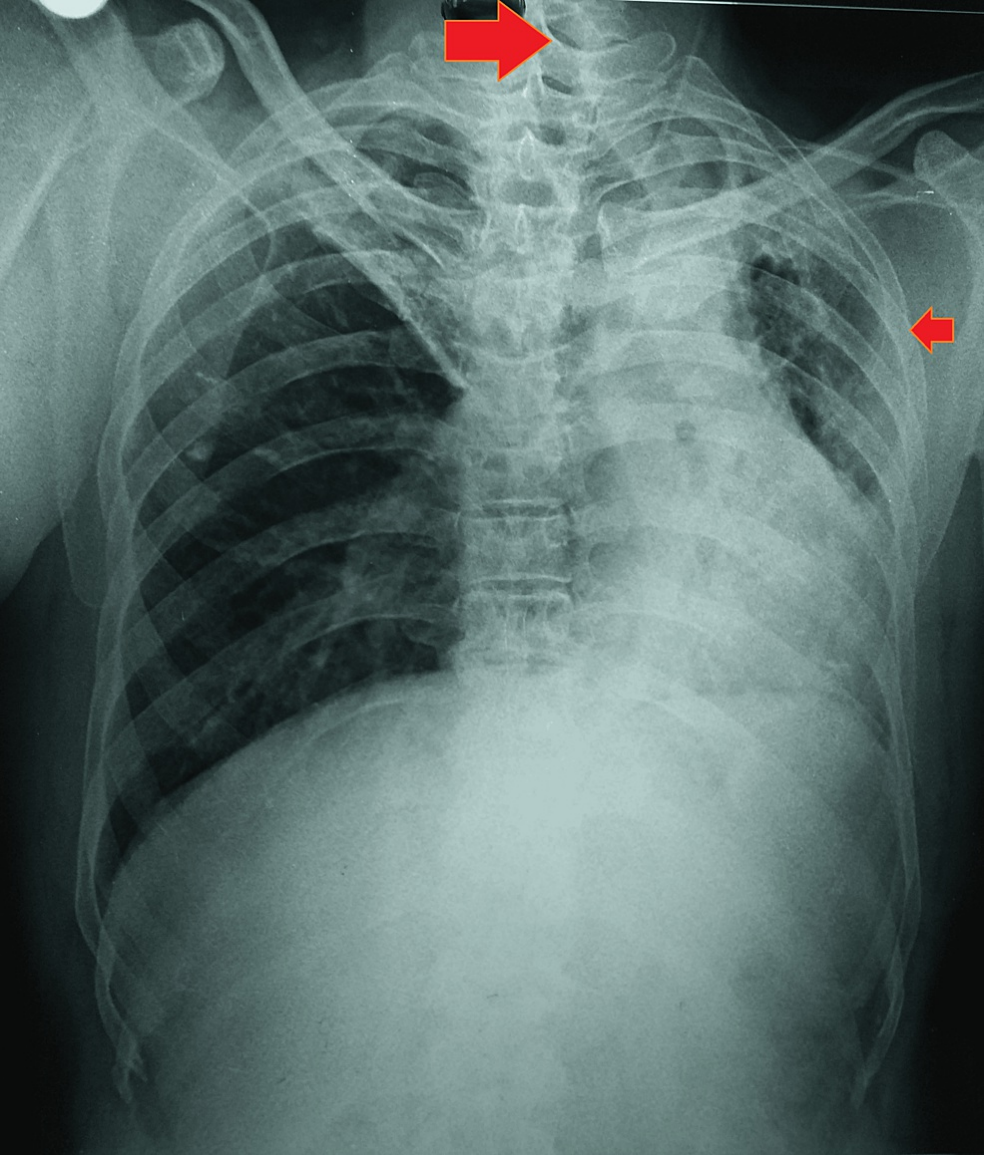
A plain chest radiograph suggestive of a destroyed left lung with tracheal deviation

A respiratory system examination revealed amphoric breathing on the left side of the chest and vesicular breath sounds on the right side, along with crepitations throughout the left lung. Other systemic examinations were unremarkable. Further diagnostic tests included a chest radiograph, which indicated fibrotic collapse of the left lung with tracheal deviation and mediastinal shift toward the left hemithorax. Rib crowding was observed on the left side, but there was no compensatory hyperinflation of the right lung (Figure 1).

Sputum examination results were negative for any pathology. The remaining laboratory investigations revealed mild anemia with a hemoglobin level of 10.8 g/dL. In addition, results from the sweat chloride test, aspergillus precipitin test, AFB-*Mycobacterium *other than tuberculosis blood panel test, thyroid profile, and routine/microscopic urine analysis were within normal ranges. The patient tested non-reactive for HIV. The pulmonary function tests indicated obstructive disease, with a forced expiratory volume in 1 second (FEV1) of 0.83 liters, a forced vital capacity (FVC) of 1.62 liters, and a modified Tiffeneau-Pinelli index (FEV1/FVC ratio) of 50%. Subsequently, a diagnosis of post-tuberculous destroyed lung was established, and the patient was prescribed a dry powder inhaler containing salmeterol plus fluticasone 250 micrograms twice daily, along with a dry powder inhaler of tiotropium 18 micrograms once daily. In addition, acebrophylline tablets at a dosage of 200 mg were recommended for nightly intake. The patient was instructed to perform breathing exercises using incentive spirometry and engage in yoga sessions. Nutritional advice comprised adopting a high-protein diet and integrating regular walking into the routine for optimal endurance. It was recommended to undergo annual influenza vaccination and administer the pneumococcal vaccine every five years. Emphasis was placed on the significance of quitting tobacco and scheduling regular check-ups at the nearest health facility during counseling sessions.

Moreover, it was observed that from the onset of tuberculosis to the identification of post-tuberculous lung destruction, the total duration spanned three years in this particular case.

## Discussion

Since 2000, an estimated 75 million individuals have survived tuberculosis, yet many will contend with post-tuberculosis lung disease, posing significant challenges to long-term respiratory health [6]. It is defined as “evidence of chronic respiratory abnormality, with or without symptoms, attributable at least in part to previous pulmonary tuberculosis.”

Post-tuberculosis lung disease results from intricate interactions among various factors, including the pathogen, host, and environmental influences. It encompasses a spectrum of conditions, such as bronchiectasis and obstructive lung disease, affecting both major and minor airways, as well as lung parenchyma, pulmonary vasculature, and pleura. These conditions are frequently compounded by co-infections and episodes of hemoptysis [7]. Those affected by post-tuberculosis lung disease face a shortened life expectancy due to respiratory or cardiovascular diseases and heightened risks of recurrent tuberculosis, although long-term outcome predictors remain elusive [7-9].

Destroyed lung syndrome is an irreversible condition characterized by complete or extensive destruction of the lung tissue, resulting in compromised lung function [4]. Oftentimes, tuberculosis (either primary or reinfection) is the contributor to this clinical condition; however, other causes are also documented [4,5].

Components of a destroyed lung syndrome are mentioned in Table 1 [3].

**Table 1:** Components of a destroyed lung syndrome Reference: Patil S et al. [3]

Components of destroyed lung syndrome
Pulmonary cavitation
Cystic bronchiectasis
Loss of lung volume
Pleuroparenchymal fibrosis
Crowding of ribs on the affected side
One-sided almost complete parenchymal anomalies
Compensatory hyperinflation on the opposite side presenting as emphysema
Mediastinal herniation

Clinical presentations of destroyed lung syndrome are predominantly chronic, although acute incidences have also been documented [10]. Patients typically present with prolonged and recurrent episodes of respiratory illnesses, characterized by persistent low-grade fever, chest tightness, purulent expectoration, dyspnea, and asthenia. Recurrent hemoptysis may complicate the clinical picture [4,10]. In acute cases, symptoms of septicemia, massive hemoptysis, or respiratory failure may also manifest [4].

According to Dhar et al., dyspnea and decreased quality of life were observed in 2,195 research participants as a result of post-tuberculosis lung damage, which resulted in a 40% drop in lung capacity [11]. Similarly, even after efficient anti-tubercular treatment, Meghi et al. found prevalent and under-recognized post-tuberculosis lung injuries in 405 study participants from Malawi, with implications for unfavorable outcomes [12]. In a study of 600 subjects from Bangladesh, 70% exhibited left lung destruction, with tuberculosis implicated in 84% of cases [4].

In 2013, George MD reported a case akin to the present case, in which a 45-year-old man was diagnosed with destroyed lung syndrome [13]. A history of tuberculosis, dyspnea, radiographic results showing mediastinal shift, and a damaged lung are among the similarities between the two cases [13]. However, there are some important differences between the two cases: the patient's age, ethnicity, the damaged lung side (left in this case), the presence of a dry cough, and the absence of dextrocardia. 

Furthermore, there is a lack of definitive treatment guidelines [14]. Conservative management typically involves symptomatic treatment using long-acting muscarinic antagonists or long-acting beta-2 agonists combined with inhaled corticosteroids to achieve bronchodilatory effects [15]. Yum et al. reported that tiotropium demonstrated therapeutic efficacy in cases of tuberculous-destroyed lungs [16]. Indacaterol was found to significantly improve forced expiratory volume in one second (FEV1) and relieve dyspnea when compared to a placebo in a recent multicenter, double-blind clinical trial [17]. In situations of destroyed lungs, surgical intervention, such as pneumonectomy, is needed to either avoid or relieve problems. However, it is considered a high-risk procedure [4]. High death rates are associated with extensive deterioration of the lungs from tuberculosis; these deaths are mainly caused by major hemoptysis, superinfections, respiratory failure, or active tuberculosis [18]. Furthermore, in patients with tuberculous-destroyed lung syndrome, hemoptysis resulting from hypertrophied bronchial arteries or Rasmussen aneurysms can cause fatal consequences [4]. Moreover, instances of damaged lung syndrome have been linked to serious consequences, including left-right shunts and septicemia/empyema [10].

The burden of chronic lung illness worldwide is largely attributed to post-tuberculosis lung diseases, including damaged lung syndrome. This emphasizes how critical it is to spread knowledge about and comprehend the psychological, social, and economic effects connected to these conditions. In order to assist clinical decision-making, inform policy, and create preventative strategies for post-tuberculosis lung illness, urgent research is necessary [12].

## Conclusions

Destroyed lung syndrome is an infrequently reported condition. With the goal of tuberculosis elimination, the majority of attention of the regulatory agencies is toward the timely diagnosis and management of tuberculosis. There is a growing number of cases related to post-tuberculosis lung diseases, such as destroyed lung syndrome. Oftentimes, due to factors, like the stigma associated with the disease, these are not reported timely, and this could result in fatal outcomes. Clinicians should have an eye for these complications, especially in high-burden settings. Moreover, large-scale data related to this clinical condition is essential for designing policies specific to timely management and preventing adverse outcomes.

## References

[1] Barberis I, Bragazzi NL, Galluzzo L, Martini M (2017). The history of tuberculosis: from the first historical records to the isolation of Koch's bacillus. J Prev Med Hyg.

[2] (2024). World health organization: global tuberculosis report 2022. https://www.who.int/teams/global-tuberculosis-programme/tb-reports/global-tuberculosis-report-2022.

[3] Patil S, Narkar S, Raka V, Dahiphale J, Choudhari S, Gondhali G (2023). ‘Destroyed lung’ as post tuberculosis sequel: a preventable stigma of ‘disease of concern’ of millennium!. Saudi J Med.

[4] Yadav S (2023). Destroyed lung syndrome in a young indian male: a case report. Cureus.

[5] Ruan H, Liu F, Li Y (2022). Long-term follow-up of tuberculosis-destroyed lung patients after surgical treatment. BMC Pulm Med.

[6] (2024). World health organization. tuberculosis fact sheet. https://www.who.int/en/news-room/fact-sheets/detail/tuberculosis.

[7] Allwood BW, Byrne A, Meghji J, Rachow A, van der Zalm MM, Schoch OD (2021). Post-tuberculosis lung disease: clinical review of an under-recognised global challenge. Respiration.

[8] Romanowski K, Baumann B, Basham CA, Ahmad Khan F, Fox GJ, Johnston JC (2019). Long-term all-cause mortality in people treated for tuberculosis: a systematic review and meta-analysis. Lancet Infect Dis.

[9] Ranzani OT, Rodrigues LC, Bombarda S, Minto CM, Waldman EA, Carvalho CR (2020). Long-term survival and cause-specific mortality of patients newly diagnosed with tuberculosis in São Paulo state, Brazil, 2010-15: a population-based, longitudinal study. Lancet Infect Dis.

[10] Genovés Crespo M, Agustín Martínez F, Callejas González FJ (2016). Destroyed lung complicated with empyema. Imaging Med.

[11] Dhar R, Singh S, Talwar D (20197). Bronchiectasis in india: results from the european multicentre bronchiectasis audit and research collaboration (embarc) and respiratory research network of india registry. Lancet Glob Health.

[12] Meghji J, Lesosky M, Joekes E (2020). Patient outcomes associated with post-tuberculosis lung damage in Malawi: a prospective cohort study. Thorax.

[13] George MD (2014). Radiological features of right destroyed lungs syndrome with pathologic dextrocardia. Int J Trop Dis Health.

[14] van Kampen SC, Wanner A, Edwards M, Harries AD, Kirenga BJ, Chakaya J, Jones R (2018). International research and guidelines on post-tuberculosis chronic lung disorders: a systematic scoping review. BMJ Glob Health.

[15] Rhee CK, Yoo KH, Lee JH (2013). Clinical characteristics of patients with tuberculosis-destroyed lung. Int J Tuberc Lung Dis.

[16] Yum HK, Park IN (2014). Effect of inhaled tiotropium on spirometric parameters in patients with tuberculous destroyed lung. Tuberc Respir Dis (Seoul).

[17] Kim CJ, Yoon HK, Park MJ (2017). Inhaled indacaterol for the treatment of COPD patients with destroyed lung by tuberculosis and moderate-to-severe airflow limitation: results from the randomized INFINITY study. Int J Chron Obstruct Pulmon Dis.

[18] Katoto PD, Musole P, Maheshe G, Bamuleke B, Murhula A, Balungwe P, Byamungu LN (2020). A miner with no left lung: extensive pulmonary destruction in delayed effective multi-drug-resistant tuberculosis treatment. Respir Med Case Rep.

